# DCZ0415, a small‐molecule inhibitor targeting TRIP13, inhibits EMT and metastasis via inactivation of the FGFR4/STAT3 axis and the Wnt/β‐catenin pathway in colorectal cancer

**DOI:** 10.1002/1878-0261.13201

**Published:** 2022-03-07

**Authors:** Sumit Agarwal, Farrukh Afaq, Prachi Bajpai, Hyung‐Gyoon Kim, Amr Elkholy, Michael Behring, Darshan Shimoga Chandrashekar, Sameer Al Diffalha, Moh’d Khushman, Shajan P. Sugandha, Sooryanarayana Varambally, Upender Manne

**Affiliations:** ^1^ Department of Pathology University of Alabama at Birmingham AL USA; ^2^ O’Neal Comprehensive Cancer Center University of Alabama at Birmingham AL USA; ^3^ Department of Medicine Division of Medical Oncology University of Alabama at Birmingham AL USA; ^4^ Department of Medicine Division of Gastroenterology University of Alabama at Birmingham AL USA

**Keywords:** colorectal cancer, DCZ0415, FGFR4, granzyme B, metastasis, TRIP13

## Abstract

Thyroid receptor‐interacting protein 13 (TRIP13), a protein of the AAA‐ATPase family, is upregulated in various human cancers, including colorectal cancer (CRC). This study focused on the inhibition of TRIP13‐induced CRC progression and signalling by DCZ0415, a small molecule targeting TRIP13. It demonstrated potent antitumour activity in TRIP13‐deregulated cancer cell lines, regardless of their p53, KRAS, BRAF, epidermal growth factor receptor or microsatellite instability status. The treatment of CRC cells with DCZ0415 resulted in decreased cell proliferation, induced cell cycle arrest in the G2‐M phase and increased apoptosis. DCZ0415 diminished xenograft tumour growth and metastasis of CRC in immunocompromised mice. DCZ0415 reduced expression of fibroblast growth factor receptor 4 (FGFR4), signal transducer and activator of transcription 3 (STAT3), and proteins associated with the epithelial‐mesenchymal transition and nuclear factor kappa B (NF‐κB) pathways in cells and xenografts exhibiting high expression of TRIP13. Additionally, DCZ0415 decreased cyclin D1, β‐catenin and T‐cell factor 1, leading to the inactivation of the Wnt/β‐catenin pathway. In a syngeneic CRC model, DCZ0415 treatment induced an immune response by decreasing PD1 and CTLA4 levels and increasing granzyme B, perforin and interferon gamma. In sum, DCZ04145 inhibits the TRIP13–FGFR4–STAT3 axis, inactivates NF‐κB and Wnt/β‐catenin signalling, activates antitumour immune response and reduces the progression and metastasis of CRC. This study provides a rationale to evaluate DCZ0415 clinically for the treatment of a subset of CRCs that exhibit dysregulated TRIP13 and FGFR4.

## Graphical abstract

TRIP13 is upregulated in patients with colorectal cancer (CRC). Here we investigated the effect of the TRIP13‐specific inhibitor DCZ0415 and observed that it blocked the TRIP13/fibroblast growth factor receptor 4 (FGFR4)/signal transducer and activator of transcription 3 axis, epithelial–mesenchymal transition, nuclear factor kappa B and Wnt/β‐catenin signalling pathways. Thus, DCZ0415 may have a potential role in treating a subset of patients with CRC that exhibit dysregulated TRIP13 and FGFR4 expression.

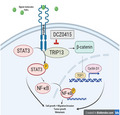

**Abbreviations**
CRC: colorectal cancer
CTLA4: cytotoxic T‐lymphocyte‐associated protein 4
EGFR: epidermal growth factor receptor
EMT: epithelial–mesenchymal transition
FGFR4: fibroblast growth factor receptor 4
IFNγ: interferon gamma
MSI: microsatellite instable
NF‐κB: nuclear factor kappa B
NSG: NOD/SCID/IL2γ receptor‐null
NT: non‐targeting
PD1: programmed cell death protein 1
STAT3: signal transducer and activator of transcription 3
TCF1: T‐cell factor 1

## 1 Introduction

Thyroid receptor‐interacting protein 13 (TRIP13) is a member of the highly conserved AAA‐ATPase family [1]. TRIP13 regulates spindle assembly checkpoint and DNA repair pathways by promoting non‐homologous end joining [2, 3], which accounts for the chromosomal instability in most human cancers [4]. TRIP13, which is involved in mitotic checkpoint regulation [5], is located at chromosome 5p15, a region that is frequently amplified in lung cancers [6, 7] and cervical carcinomas [8]. The abnormal expression of TRIP13 is also implicated in chemoresistance [9]. Further, the activation of TRIP13 induces radiation resistance but improves sensitivity to cetuximab [10]. Thus, TRIP13 may be a therapeutic target for human cancers.

Our recent study [11] and others [12, 13, 14, 15, 16] have found that TRIP13 is upregulated in colorectal cancers (CRCs; 55–80%), promotes cancer cell growth, promotes disease progression and indicates poor patient survival. Our previous study [11] also showed that, in CRC cell lines, the overexpression of TRIP13 drives metastasis through epidermal growth factor receptor (EGFR) activation, interaction with fibroblast growth factor receptor 4 (FGFR4), activation of the Wnt signalling pathway and the epithelial–mesenchymal transition (EMT), regardless of cell p53, KRAS, BRAF and microsatellite instability (MSI) status, suggesting that it is a potential target for the treatment of CRC.

Fibroblast growth factor receptor 4, a receptor tyrosine kinase belonging to the FGFR family, is located on the long arm of chromosome 5 (5q35.1). FGFR4 and its ligand (FGF19) are overexpressed in various cancers, including CRCs [17]. Amplification and overexpression of FGFR4 are observed in hepatocellular carcinoma [18], lung squamous cell carcinoma [19], prostate cancer [20], breast cancer [21] and thyroid cancer [22]. In cancers, the hyper‐activation of FGFR4 signalling is a common alteration leading to the activation of various intracellular pathways, including GSK3β/β‐catenin/E‐cadherin [23], FGFR4–GSK3b–Nrf2 signalling [24] and PI3K‐AKT [25]. These results indicate that dysregulation of FGFR4 could be a common event that elevates oncogenic signalling during tumour development. The involvement of FGFR4 in cellular mechanisms of tumour aggressiveness suggests its driver role in cancer and consequently its potential as a therapeutic target. Therefore, inhibiting the activation of growth factor receptors, especially FGFR4, may be a promising strategy for the treatment of CRC. Since our prior study demonstrated an interaction of FGFR4 with TRIP13 in the CRC cell lines, SW480 and HT29 [11], we sought to determine the downstream signalling pathways that lead to TRIP13‐driven CRCs by targeting TRIP13 with a small‐molecule inhibitor.

Recently, an inhibitor specifically targeting TRIP13, DCZ0415, was developed and tested in myeloma experimental models and in primary cells derived from drug‐resistant myelomas [26]. This prior study confirmed the binding of DCZ0415 to TRIP13 by conducting pull‐down, nuclear magnetic resonance spectroscopy and surface plasmon resonance–binding assays. Furthermore, this study showed that the treatment of immunocompetent myeloma models with DCZ0415 increases the numbers of immune cells (CD3, CD4 and CD8) and inhibits nuclear factor kappa B (NF‐κB) activity, suggesting the immunotherapeutic value of inhibiting TRIP13 [26].

In our prior study [11], we demonstrated the oncogenic role of TRIP13 by evaluating tumour growth and metastasis of CRC. In the present study, we evaluated TRIP13 as a potential therapeutic target by treating a panel of CRC cells exhibiting various molecular alterations in p53, KRAS, BRAF, EGFR and MSI, and xenograft models with DCZ0415, and showed that DCZ0415 inhibits CRC cell proliferation and tumour growth and progression. Additionally, the findings provided insights into the mechanism of CRC progression through the TRIP13/FGFR4/signal transducer and activator of transcription 3 (STAT3) axis. Blocking of this axis, EMT, NF‐κB and Wnt/β‐catenin signalling by DCZ0415 reduced CRC progression, indicating that DCZ0415 is a potential inhibitor of CRCs exhibiting TRIP13 overexpression and suggests a valid future therapeutic strategy for CRC tumours overexpressing TRIP13.

## 2 Methods

### 2.1 Human tissue specimens

For the present study, the Tissue Biorepository of the University of Alabama at Birmingham (UAB) collected frozen cancer tissue specimens and their corresponding adjacent non‐cancerous/benign tissues, from CRC patients after obtaining approval from the UAB institutional review board and ethics committee (IRB#090513004). This study was a retrospective protocol that was exempt from participant consent. Frozen tissue specimens were utilized for examination of protein expression by western blot analyses. The study methodologies conformed to the standards set by the Declaration of Helsinki.

### 2.2 Cell lines, RNA interference and DCZ0415

#### 2.2.1 CRC cell lines

**Fig. 1:**
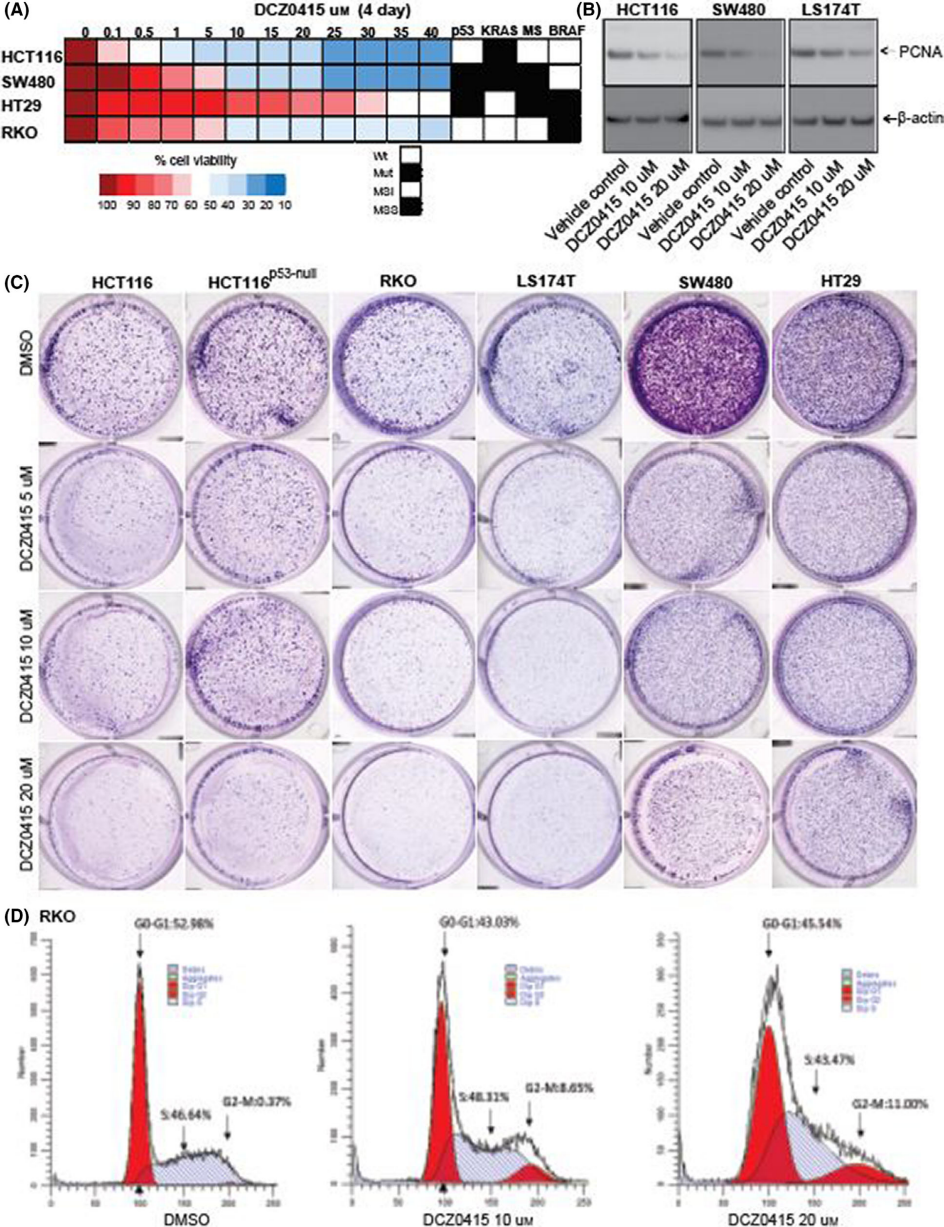
Pharmacologic inhibition of DCZ0415 decreases malignant phenotypes of CRC. (A) MTT assay showing viability of CRC cells exhibiting various genetic alterations after treatment with serially diluted DCZ0415 concentrations. The heat map was plotted to show the viability of CRC cells after treatment with DCZ0415 at 4 days. (B) PCNA protein expression was analysed in CRC cells after treatment with DCZ0415 for 48 h. (C) Colony formation assay with CRC cells treated with 5, 10 or 20 µm DCZ0415. (D) Cell cycle analysis was performed in CRC cells after treatment with DCZ0415 for 24 h. The experiments had three replicates.

We studied the inhibitory effect of DCZ0415 and the functional role of TRIP13 in CRC cell lines (HCT116, LS174T, RKO, SW480 and HT29) that exhibit various combinations of mutational status of *p53*, *KRAS* and *BRAF*; expression of EGFR; and MSI as shown in Fig. 1A. We included two versions of HCT116 cells—one exhibiting wild‐type p53 (HCT116) and another with the p53 gene homozygosity disrupted, HCT116^p53‐null^ [27]. These human cell lines were purchased from American Type Culture Collection (Manassas, VA, USA) and were grown in McCoy’s media (Corning™ Cellgro™, Fisher Scientific Co., Pittsburgh, PA, USA) supplemented with 10% FBS (Invitrogen, ThermoFisher Scientific, Carlsbad, CA, USA) and penicillin‐streptomycin. MC38, mouse colon cancer cell line, was a gift from P. Datta, Department of Medicine, UAB. These cells were grown in RPMI‐1640 medium. The UAB Heflin Center for Genomic Sciences performed cell line identification and authentication by short tandem repeat DNA profiling. Mycoplasma screening was performed regularly.

To generate lentiviruses, a pGreenPuro™ shRNA expression lentivector having DNA sequences against TRIP13 (Systembio, Palo Alto, CA, USA) was established by the UAB Neuroscience NINDS Vector Core (P30 NS047466). TRIP13 shRNA sequences are given in Table S1. CRC cells were infected with lentiviruses expressing TRIP13 shRNA or non‐targeting (NT) shRNA with 2 μg·mL^−1^ polybrene. Puromycin (1 μg·mL^−1^; Life Technologies, ThermoFisher Scientific) was used for the selection of stable cell lines as described previously [28, 29]. Selected cells were used for immunoprecipitation analyses and cell‐based functional assays.

DCZ0415, an inhibitor of TRIP13, was procured from MedChemExpress (Cat. No# HY‐130603, Monmouth Junction, NJ, USA) and was prepared according to the vender’s suggestions. In brief, for cell culture experiments, it was dissolved in DMSO, but, for animal experiments, it was dissolved in 10% DMSO, 40% PEG300, 5% Tween 80% and 45% saline.

### 2.3 Immunoblot analyses

Immunoblot analyses were used to evaluate protein expression in CRC cells and tissues as described earlier [28, 30]. Briefly, lysates were prepared in NP‐40 buffer (Cat#BP‐119, Boston BioProducts, Ashland, MA, USA) with HALT protease and phosphatase inhibitors cocktail (Cat# 78440; ThermoFisher Scientific), followed by a brief sonication under cold conditions. NuPAGE™ 4–12% Bis‐Tris Midi Protein Gels, 20‐well SDS/PAGE, were used to separate proteins (ThermoFisher Scientific). Protein samples were transferred onto Immobilon‐P PVDF membranes (EMD Millipore, Billerica, MA, USA). Blocking buffer [Tris‐buffered saline, 0.1% Tween (TBS‐T), 5% non‐fat dry milk] was used to block non‐specific binding sites, followed by overnight incubation with primary antibodies at 4 °C and incubation with horseradish peroxidase‐conjugated secondary antibody for 1 h. Signals were visualized by Luminata™ Crescendo chemiluminescence western blotting substrate according to the manufacturer’s protocol (EMD Millipore, Billerica, MA, USA). A list of primary antibodies, along with their dilutions, catalogue number and company details, is provided in Table S2.

### 2.4 Cell viability assay

After treating cells with DCZ0415, their viability was measured by MTT (3‐(4,5‐dimethylthiazol‐2‐yl)‐2,5‐diphenyltetrazolium bromide; catalog#M5655; Sigma Aldrich) assay. Living cells reduced MTT to a pigment, formazon, which was measured in a Synergy™ HTX Multi‐Mode Microplate Reader (BioTek, Winooski, VT, USA) at 540 nm after 2–2.5 h of incubation. Changes in cell viability were assessed by normalizing the absorbance of DCZ0415‐treated cultures with the untreated control cultures.

### 2.5 Cell cycle analysis

Colorectal cancer cells were seeded in six‐well plates at a density of 5 × 10^5^/mL and then treated with DCZ0415 at various concentrations for 24 h. The cells were fixed with cold 70% ethyl alcohol at 4 °C overnight and then washed with PBS, followed by incubation with RNase A for 30 min. Propidium iodide staining was performed for 30 min at room temperature. Cell cycle analysis was accomplished with a BD LSR Fortessa™ Flow Cytometer (BD Biosciences, Mountain View, CA, USA).

### 2.6 Colony formation assay

Colony formation assays were performed as described previously [29]. CRC cells (1 × 10^3^) were seeded into six‐well plates in triplicate for overnight, followed by the treatment the next day. After 10 days of incubation, cells were fixed with 5% glutaraldehyde and stained with crystal violet (Sigma‐Aldrich). An Amersham Imager 600RGB (GE Healthcare Life Sciences, Pittsburgh, PA, USA) was used to take images of six‐well plates with colonies.

### 2.7 Quantification of apoptosis

Apoptotic cells were determined by TACS® Annexin V‐FITC apoptosis detection kit, obtained from R&D System (Minneapolis, MN, USA), as per manufacturer's protocol. Briefly, CRC cells were grown at a density of 1 × 10^6^ cells in 100‐mm culture dishes and were treated with DCZ0415 (10 and 20 μm) for 48 h. Thereafter, the cells were trypsinized and harvested by centrifugation at 500 ***g*** for 10 min and then washed with ice‐cold PBS. Approximately 5 × 10^5^ cells were suspended in 400 μL of 1× Annexin V binding buffer, 1 μL of Annexin V‐FITC and 10 μL of propidium iodide. The cells were then incubated at room temperature for 15 min in the dark and analysed by LSR Fortessa.

### 2.8 Invasion assay

As described previously [30], Corning BioCoat™ Matrigel matrix (Corning, New York, NY, USA) plates were used to perform invasion assays for CRC cells treated with DCZ0415 for 24 h. Treated cells (5 × 10^4^) in 500 μL of serum‐free medium were layered onto 8‐μm pore inserts in 24‐well plates in triplicate; 10% FBS was added to the lower chambers. After 48 h, non‐invading cells and the Matrigel matrix were removed from the front of the insert with a cotton swab. Invaded cells, on the bottom of insert, were fixed with 5% glutaraldehyde and stained with crystal violet. Images were taken with a phase‐contrast microscope.

### 2.9 Wound healing assay

Colorectal cancer cells (1 × 10^6^) were seeded onto 35‐mm Petri dishes for overnight as described earlier [31]. Next day, an artificial wound was created on the confluent cell monolayers using tips of 200‐μL pipets. Floating cells were washed out of the lanes with PBS, and images were taken at this point (considered as 0 h) with an inverted phase‐contrast microscope with a 4× objective. For inhibitor studies, cells were treated with DCZ0415 after the first image was taken. Images were taken again at 24 h.

### 2.10 RNA extraction and real‐time quantitative PCR

Total RNA was isolated from CRC cells after 24 h of DCZ treatment (10 and 20 µm) along with DMSO control, with TRIzol reagent using the manufacturer's instructions (Invitrogen). Total RNA (2 µg) was reverse transcribed using High capacity cDNA reverse transcription kit with RNase inhibitor (Applied Biosystems, Thermo Fisher Scientific, Waltham, MA, USA). Ten nanograms of cDNA was used for validation and quantification of EMT marker genes using PowerUp SYBR green master mix (Applied Biosystems, Thermo Fisher Scientific) on an ABI real‐time PCR machine and analysed using Quant‐studio Real‐Time PCR software (Applied Biosystems). The primer sequences are provided in Table S3.

### 2.11 Tumour xenograft and metastasis model

NOD/SCID/IL2γ receptor‐null (NSG) mice (6–8 weeks old) were obtained from the Jackson Laboratory (Farmington, CT, USA) and housed in animal facility of UAB under pathogen‐free conditions with 12‐h light/12‐h dark schedule at 24 ± 2 °C temperature and 50 ± 10% relative humidity. All animal studies were approved by the Institutional Animal Care and Use Committee of UAB (IACUC‐21501). For tumour xenograft experiments, both male and female NSG mice(*n* = 5 for each group) were implanted with 1 × 10^6^ CRC cells in 100 μL of 50% Matrigel subcutaneously at the flank as described earlier [32, 33]. When the tumours reached 100–150 mm^3^, animals were randomized into groups of five and dosed intraperitoneally every other day. Growing tumours were palpated, and calipers were used to measure tumour sizes. Tumour volumes were calculated by the formula *V* = ½ (length × width^2^).

For metastasis studies, luciferase‐tagged CRC cells (0.5 × 10^6^ in 100 µL of media without FBS) were injected into the lateral tail veins of 6‐ to 8‐week‐old one female and one male NSG/SCID/IL2γ mice (*n* = 2 for each group; the Jackson Laboratory), as described previously [31, 34]. Metastases were monitored by bioluminescent imaging with an *In Vivo* IVIS Lumina Series III spectrum imaging system (Perkin Elmer, Waltham, MA, USA). d‐Luciferin (100 μL of 10 mg·mL^−1^ dissolved in PBS) was injected into mice intraperitoneally at 10 min before luminescence imaging. At the end of the experiment, mice were euthanized and their organs were removed for the assessment of metastases.

### 2.12 Syngeneic mouse models

To examine the effect of DCZ0415 on immunocompetent mice, murine CRC cells, MC38 (0.25 × 10^6^) were injected into male and female C57BL/6 mice obtained from the Jackson Laboratory, and, when the median tumour size reached 100–150 mm^3^, animals were grouped into vehicle (*n* = 5) and DCZ0415 (*n* = 5) treatment arms. DCZ0415 (25 mg·kg^−1^) was injected intraperitoneally every day for 12 days. The animals were sacrificed when tumour volumes reached 2500 mm^3^ or other termination criteria applied (e.g. ulceration, body weight loss > 20%) in accordance with the approved IACUC‐21501 protocol. After obtaining RNA samples from these tumours, quantitative PCR (qPCR) for programmed cell death protein 1 (PD1) and cytotoxic T‐lymphocyte‐associated protein 4 (CTLA4) was performed and protein lysates were probed for granzyme B, perforin and interferon gamma (IFNγ). The primer details are provided in Table S3.

### 2.13 Immunohistochemical analysis

The immunophenotypic expression of CD3, CD4 and CD8 in tumours collected from immunocompetent mice was assessed on tissue sections by immunohistochemical (IHC) assays as described earlier [11]. In brief, after de‐paraffinization, rehydration, antigen retrieval by EDTA buffer and blocking with horse serum, primary antibodies (Table S2) were used to probe tumour sections. Next, sections were incubated with ImmPRESS HRP anti‐mouse IgG (Cat# MP‐7402, RR ID: AB_2336528, Vector laboratories, Burlingame, CA, USA), as a secondary antibody, was applied for 1 h, and immunoreactivity was assessed by diaminobenzidine (Cat# SK‐4100, RR ID: AB_2336382; Vector Laboratories). Hematoxylin QS (Cat#H‐3404; Vector Laboratories) was used for nuclear staining. After rehydration, mounting was accomplished with VectaMount Permanent Mounting Medium (Cat#H‐5000; Vector Laboratories).

### 2.14 Statistical analysis

A student *t*‐test *P*‐value < 0.05 was considered statistically significant. Data were expressed as means ± standard deviation for triplicate.

## 3 Results

### 3.1 DCZ0415, a TRIP13‐specific inhibitor, decreases malignant phenotypes of CRC

We investigated the inhibitory effect of DCZ0415 on CRC cells (RKO, LS174T, SW480 and HT29) by MTT cell viability assays. In all tested cell lines, cell viability was lowered in a concentration‐dependent manner by DCZ0415, as compared to vehicle control, after treating for 2 (Fig. S1A) and 4 days (Fig. 1A). Moreover, the treatment of CRC cells with DCZ0415 reduced the protein expression of PCNA, a well‐established marker of cell proliferation, which further validates the MTT assay findings (Fig. 1B).

We next evaluated the effect of DCZ0415 on colony formation of CRC cells; DCZ0415 reduced the colony‐forming capacity of these cells (Fig. 1B). Findings of assays that assessed the effect of DCZ0415 on cell invasion showed that DCZ0415 suppressed CRC cell invasion (Fig. S1B). In addition, we analysed the effect of DCZ0415 treatment on the morphology of CRC cells. We found that DCZ0415‐treated CRC cells showed less spindle shaped appearance, became round, and detached from the surface of the plate when compared to untreated cells (Fig. S1C). These results show that inhibition of TRIP13 by DCZ0415 reduced the malignant phenotypes of CRC cells regardless of their *p53, KRAS*, BRAF, EGFR and MSI status. Furthermore, cell cycle analysis was used to assess the inhibitory effect of DCZ0415 on RKO CRC cells. After 24 h of treatment with DCZ0415 at either of two concentrations (10 or 20 µm), cell numbers at the G2/M phase were elevated, and there were lower cell numbers at the G0/G1 phase (Fig. 1C).

### 3.2 DCZ0415 reduces FGFR4/STAT3/NF‐κB levels and induces apoptosis in CRC cells

**Fig. 2:**
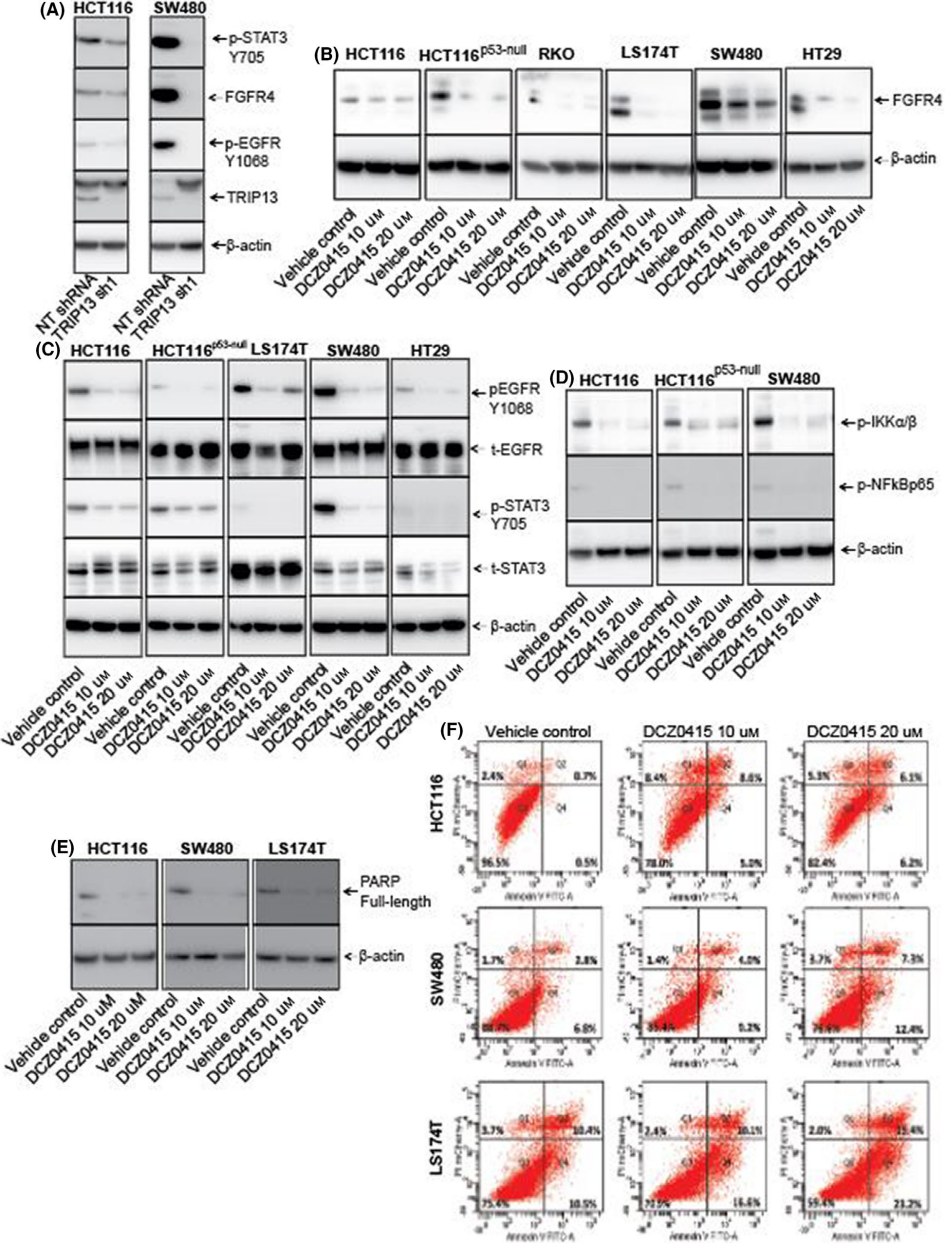
DCZ0415 inhibits the downstream signalling pathway of TRIP13. (A) Western blot analysis of TRIP13, p‐EGFR, FGFR4 and p‐STAT3 protein expressions in CRC cells transfected with TRIP13 shRNA. (B) Western blot analysis showing the levels of FGFR4 in lysates of CRC cells treated with DCZ0415. (C) For various cells, the levels of p‐STAT3, t‐STAT3, p‐EGFR and t‐EGFR, determined by western blot analysis, were assessed after CRC cells were exposed to DCZ0415. (D) For various cells, the levels of p‐IKKα/β and p‐NF‐κBp65, determined by western blot analysis, were assessed after CRC cells were exposed to DCZ0415. (E) PARP protein expression in DCZ0415‐treated CRC cells. (F) Apoptosis was quantified by Annexin V/PI staining after the treatment of CRC cells with DCZ0415 (10 and 20 µm) for 48 h. The experiments had three replicates.

FGFR4 signalling is implicated in CRC development and progression [17]. Since our prior study [11] indicated that TRIP13 interacts with FGFR4 in CRC cells and suggested that this interaction was involved in CRC progression, we evaluated DCZ0415‐mediated inhibition of FGFR4 and downstream signalling in CRC cells. TRIP13 knockdown in HCT116 and SW480 cells decreased the levels of FGFR4 (Fig. 2A). Levels were also lower in lysates of DCZ0415‐treated (Fig. 2B) CRC cell lines. The overexpression of FGFR4 secretes amphiregulin, a ligand of EGFR that consequently activates EGFR [35]. Aberrant activation of EGFR and its marked phosphorylation have been shown to increase cell proliferation, invasion, metastasis and development of resistance to various treatments in cancers, including CRC [36, 37]. In our previous study [11], we demonstrated that TRIP13 knockdown reduces the activation of EGFR. Now, we validated the previous findings of lower levels of p‐EGFR in lysates of TRIP13 knockdown (Fig. 2A) and DCZ0415 (Fig. 2C)‐treated CRC lysates.

Since, in CRCs, FGFR4 mediates cancer progression via phosphorylation of STAT3 [38], we assessed its phosphorylation status at the Y705 site in CRC cells after TRIP13 shRNA knockdown and treatment with DCZ0415. TRIP13 knockdown (Fig. 2A) and treatment with DCZ0415 (Fig. 2C) decreased the phosphorylation of STAT3 at the Y705 site. Since, in cancers, the NF‐кB pathway and activation of STAT3 are closely linked [39, 40], we evaluated the activation of NF‐κB and the IKKα/β status in CRC cells treated with DCZ0415. In CRC cells treated with DCZ0415, there were lower expression levels of phosphorylated (p)‐NF‐κBp65 and phosphorylated (p)‐IKKα/β as compared to untreated control cells (Fig. 2D). Since inhibition of STAT3 signalling induces apoptosis via caspase activation and cleavage of poly(ADP‐ribose)polymerase (PARP) [41], we determined if DCZ0414 induces apoptosis in CRCs. Western blotting demonstrated that CRC cells treated with DCZ0415 had decreased expression of full‐length PARP protein, an indicator of apoptosis (Fig. 2E). Furthermore, staining with Annexin V‐FITC and propidium iodide was used to confirm apoptosis induction on CRC cells by DCZ0415. As compared with the control cells, the features of apoptotic cell death, including the induction of early (Annexin V‐FITC^+^/PI^−^) and late‐stage (Annexin V‐FITC^+^/PI^+^) apoptosis were noticed in DCZ0415‐treated CRC cells (Fig. 2F). These results elucidated that the effect of DCZ0415 treatment on the reduction of CRC cell growth was also linked with the induction of apoptosis. Thus, we conclude that DCZ0415 deactivates the TRIP13–FGFR4–STAT3 axis and the NF‐кB pathway, leading to apoptosis, which results in the reduced progression of CRC.

### 3.3 DCZ0415 reduces CRC tumour growth in xenograft models

**Fig. 3:**
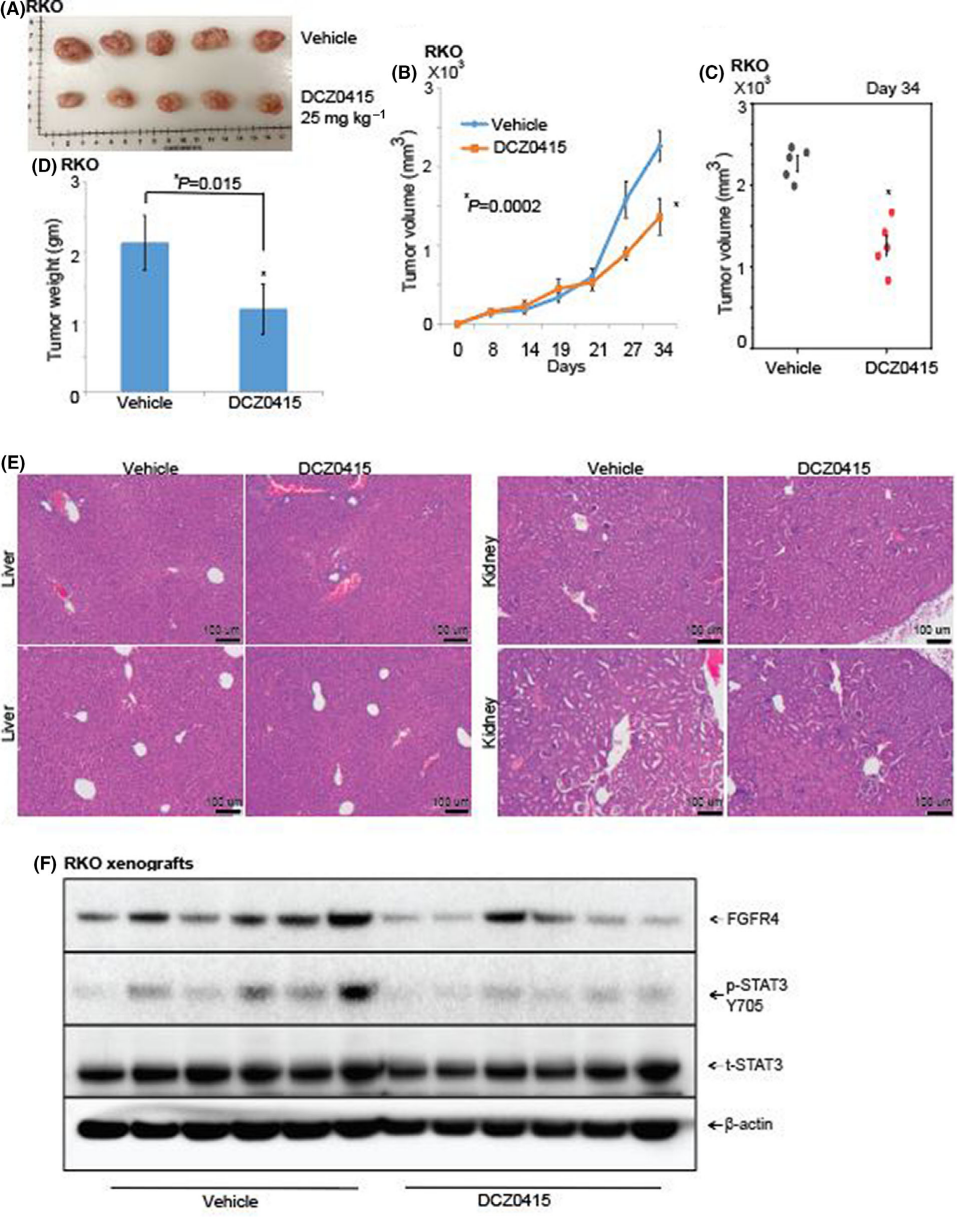
DCZ0415 reduces CRC tumour growth in immunocompromised mice. (A) Representative photograph showing tumours excised from two different experimental groups: vehicle (*n* = 5) and DCZ0415 (25 mg·kg^−1^; *n* = 5). (B) Graph showing tumour growth measured as tumour volumes for the vehicle and DCZ0415 groups (error bar indicates mean ± SD). DCZ0415 were provided to animals 12 times on alternate days after tumours reached 100–200 mm^3^ (*P = 0.0002). (C) Dot plot showing tumour growth in vehicle‐ and DCZ0415‐treated mice at day 34 (*P = 0.0002). (D) Weights of the tumours excised from the mice in each treatment group were determined at the end of the experiment on day 34 (error bar indicates mean ± SD). Asterisk (*) showing statistically significant data (*P = 0.015). (E) Representative microphotographs of the liver and kidney sections after administration of vehicle or DCZ0415 to show signs of toxicity (Scale bar = 100 µm). (F) Protein levels of FGFR4, p‐STAT3 and t‐STAT3 in lysates of xenografts treated with DCZ0415.

We next utilized xenograft models to assess the therapeutic potential of DCZ0415. CRC cells were subcutaneously injected into immunodeficient NSG mice to establish xenografts, and, after the tumours reached 100–200 mm^3^ in size, DCZ0415 was administered on alternate days for 12 times and tumour growth was assessed. Treatment with DCZ0415 reduced tumour growth in relation to those exposed to vehicle alone (Fig. 3A). Moreover, compared with the vehicle group, the tumour size (Fig. 3B,C) and weight (Fig. 3D) in the DCZ0415 treatment group were lower. Haematoxylin and eosin (H&E) staining of liver and kidney showed no signs of toxicity after administration of DCZ0415 (Fig. 3E). Moreover, western blotting assessments of FGFR4 and p‐STAT3 in xenograft tumour lysates revealed that, consistent with the *in vitro* results, DCZ0415 inhibited the expression of these proteins (Fig. 3F).

### 3.4 DCZ0415 decreases metastasis in an experimental tail‐vein model

**Fig. 4:**
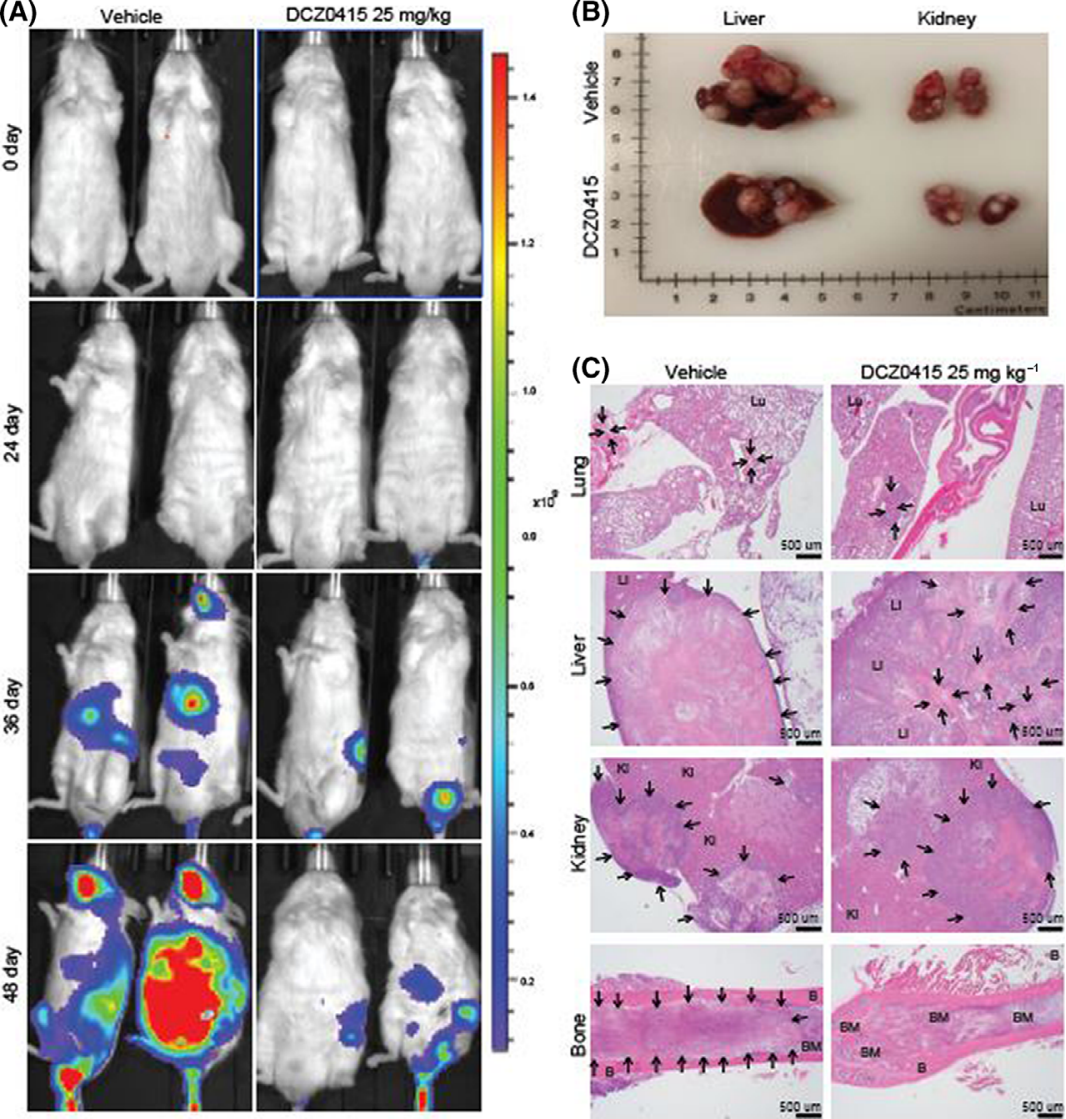
DCZ0415 reduces metastasis of CRC cells. (A) Luciferase‐transduced RKO cells were injected into the tail veins of NSG mice, which were treated with DCZ0415 (alternate days injection of 10 mg·kg^−1^ i.p.) or vehicle. (B) Representative photograph showing the liver and kidney from mice. (C) H&E images of the lung, liver, kidney and bone in vehicle‐ or DCZ0415‐treated tumours. Arrows indicate metastatic lesions; Lu—lung; Li—liver; Ki—kidney; B—bone; BM—bone marrow. 2×; scale bar, 500 µm.

We evaluated the effects of DCZ0415 on metastasis using an experimental tail‐vein model. DCZ0415 reduced distant spread of CRC cells as depicted by luciferase activity (Fig. 4A) and decreased metastases to the liver and kidney (Fig. 4B). H&E analysis showed that the organs of mice dosed with DCZ0415 harboured few metastatic tumour nodules, whereas organs of mice dosed with the vehicle were extensively occupied by metastatic tumours (Fig. 4C). These findings suggest that DCZ0415, by inhibiting TRIP13, is a potential approach for the treatment of metastatic CRC.

### 3.5 Inhibition of TRIP13 by DCZ0415 modulates the EMT and Wnt/β‐catenin signalling pathway‐associated proteins in CRC

**Fig. 5:**
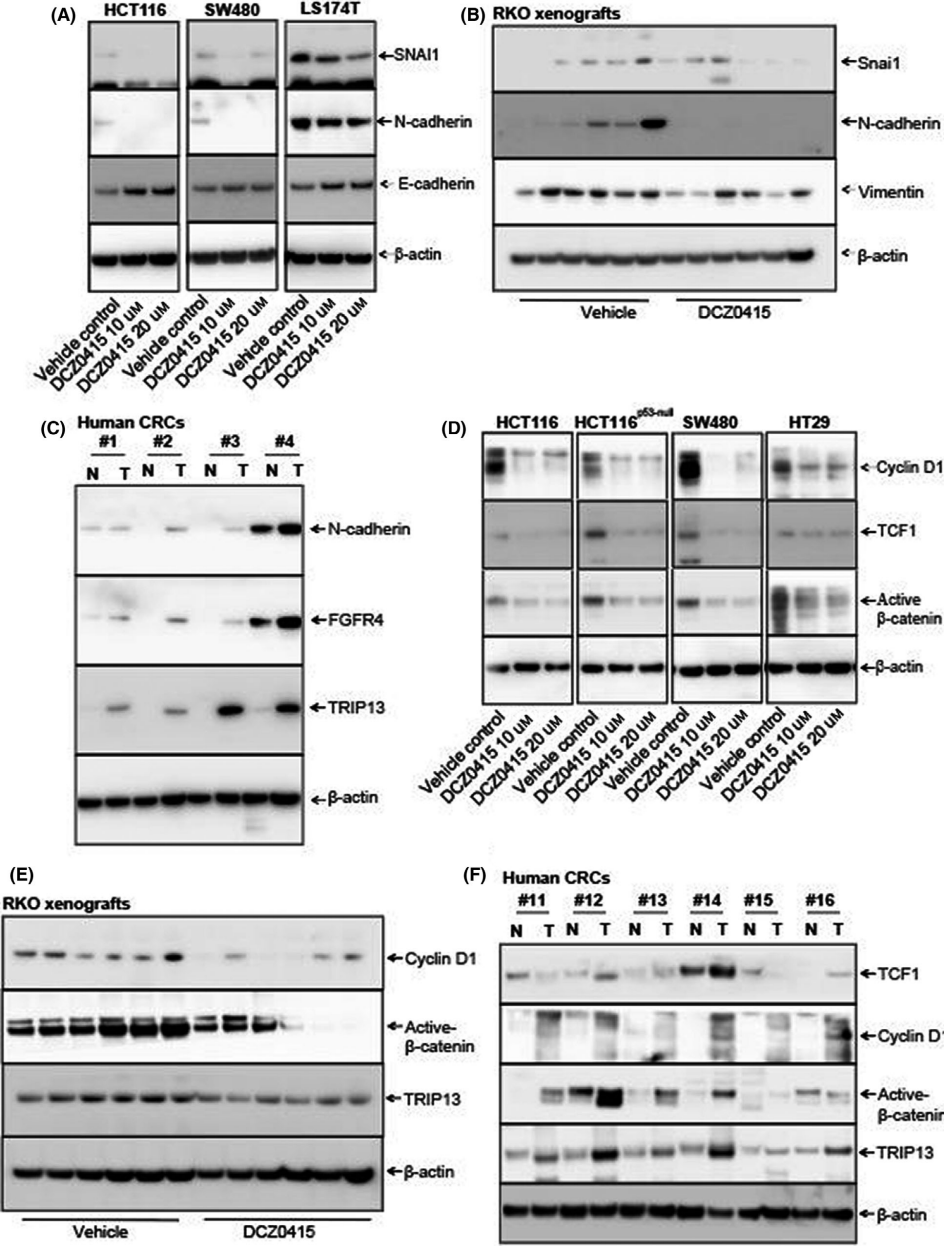
DCZ0415 modulates the EMT and Wnt signalling of CRC cells. (A) Western blot analysis showing the expression of EMT proteins in lysates of DCZ0415‐treated CRC cells. (B) Vimentin, N‐cadherin and Snai1 protein expression in DCZ0415‐treated xenografts. (C) Immunoblot analysis for TRIP13, FGFR4 and N‐cadherin levels in corresponding normal colon (N) and CRC (T) tissue samples of patients. Samples from four CRC patients were utilized for this experiment. Expression of Wnt/β‐catenin pathway target molecules in DCZ0415‐treated (D) cells and (E) xenograft lysates as determined by western blotting. (F) Immunoblot analysis for molecules of the Wnt/β‐catenin signalling pathway in corresponding normal colon (N) and CRC (T) samples.

Previously, we demonstrated that TRIP13 is involved in the CRC EMT [11]. To determine whether DCZ0415 inhibits the EMT, we used western blotting to measure changes in molecular markers of the EMT in CRC cells. DCZ0415‐treated CRC cells showed changes in the expression of EMT molecules as E‐cadherin was increased while N‐cadherin and Snai1 were decreased (Fig. 5A). We also evaluated the effects of DCZ0415 treatment on mRNA expression of EMT markers in CRC cells. Our results show increased mRNA expression of E‐cadherin (epithelial marker) with concomitantly decreased mRNA expression of N‐cadherin, vimentin and Snai1 (mesenchymal markers) in HCT116 and SW480 cells treated with DCZ0415 (Fig. S2A,B).

Furthermore, expressions of N‐cadherin, vimentin and Snai1 were lower in lysates of DCZ0415‐treated CRC xenografts compared to the controls (Fig. 5B). The expression of E‐cadherin was not detected in RKO xenografts, and this is consistent with the earlier published report that RKO cells lack E‐cadherin expression [42]. These data demonstrated that DCZ0415 inhibited the TRIP13‐induced EMT. To determine if the regulation of FGFR4 and N‐cadherin by TRIP13 is clinically relevant, we checked the expression of FGFR4 and N‐cadherin, which were downregulated by DCZ0415 treatment or TRIP13 knockdown in CRC cells, in human CRCs, which exhibited high TRIP13 expression. There was a positive correlation among TRIP13, FGFR4 and N‐cadherin expression in CRCs (Fig. 5C). These data validate the direct association between TRIP13 and FGFR4, suggesting that activation of this axis is involved in CRC progression. Our previous study showed that TRIP13 is involved in CRC progression via the Wnt/β‐catenin signalling pathway [11]. Thus, we determined if DCZ0415 treatment affected Wnt/β‐catenin signalling pathway in CRC. In lysates of DCZ0415‐treated CRC cells, there were lower expressions of β‐catenin, cyclin D1 and T‐cell facto 1 (TCF1; Fig. 5D). Moreover, in xenograft tissues, proteins of the Wnt signalling pathway were lower after mice were dosed with DCZ0415 (Fig. 5E). In addition, as a proof‐of‐principle, we evaluated, by western blotting, human CRC specimens exhibiting high TRIP13 expression for its association with Wnt/β‐catenin signalling. All tested CRCs demonstrated high levels of β‐catenin and cyclin D1 (Fig. 5F), indicating activation of Wnt/β‐catenin signalling in CRCs with high TRIP13 expression.

### 3.6 TRIP13 inhibition enhances antitumour immune response

**Fig. 6:**
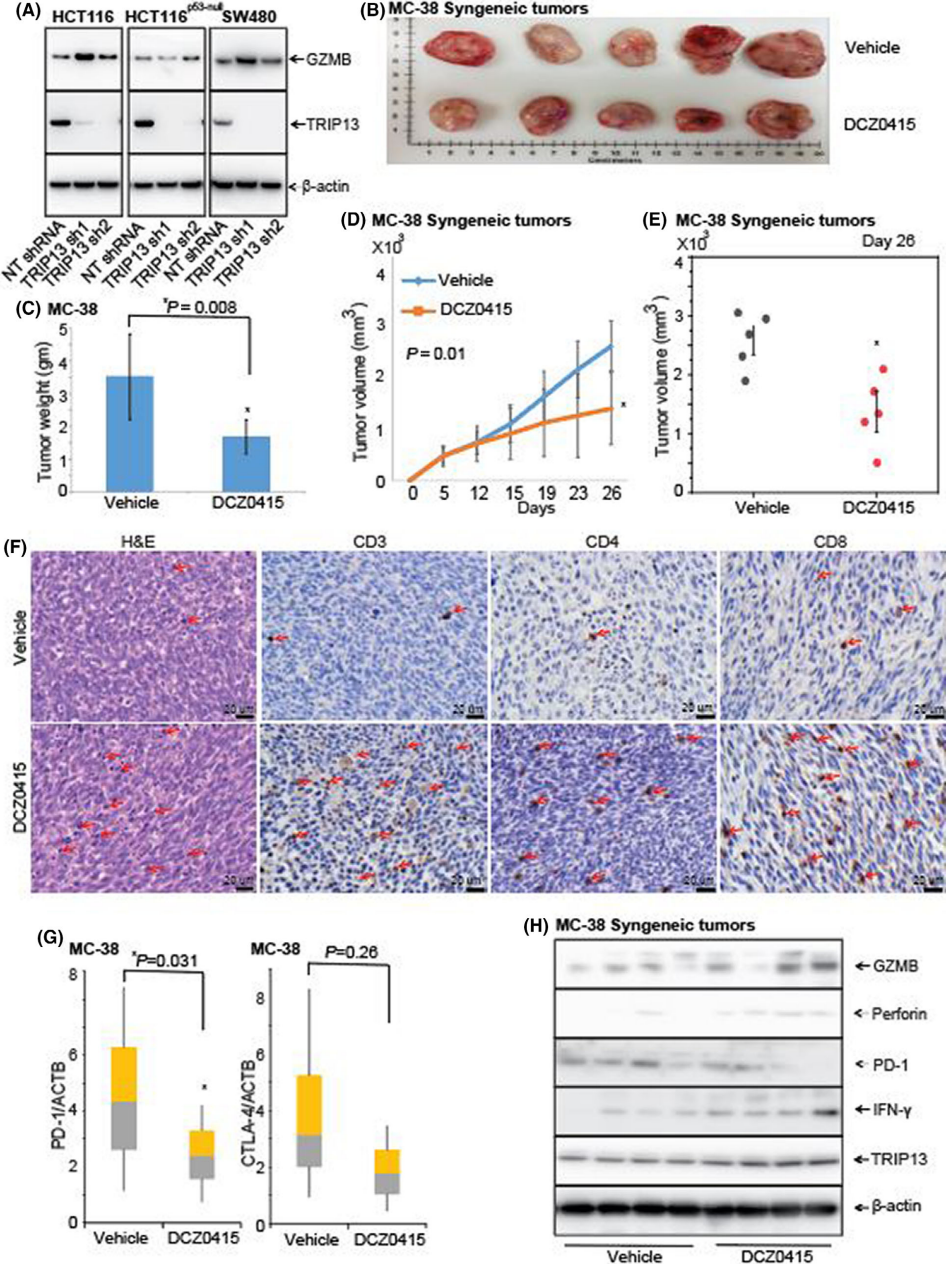
DCZ0415 increases immune infiltration in an immunocompetent model. (A) Western blot analysis to show the expression of GZMB and TRIP13 in protein lysates of CRC cells exhibiting TRIP13 knockdown (B) Photographs of syngeneic mouse tumours generated by implanting MC38 cells into C57/BL6 mice after treatment with vehicle and DCZ0415 (*n* = 5). (C) Tumour weights are shown for vehicle‐ and DCZ0415‐treated tumours (error bar indicates mean ± SD, *P = 0.008). (D) Kinetics of tumour growth treated with vehicle and DCZ0415 (error bar indicates mean ± SD, *P* = 0.01). (E) Dot plot showing tumour growth in vehicle‐ and DCZ0415‐treated mice at day 26 (error bar indicates mean ± SD, *P = 0.01). (F) H&E and IHC analysis to show the infiltration of immune cells and expression of T‐cell markers in DCZ0415‐treated immunocompetent tumours, Scale bar‐ 20 µm. Arrows indicate positive staining. (G) qPCR analysis to show the levels of PD1 and CTLA4 in DCZ0415‐treated tumours, relative to vehicle control (error bar indicates mean ± SD; *P* = 0.031 and *P* = 0.26 respectively). (H) Protein levels of GZMB, perforin, PD1, IFN‐γ and TRIP13 are shown in vehicle‐ and DCZ0415‐treated tumours.

To investigate the relation of TRIP13 with immune response, we investigated the RNA sequencing data of TRIP13 knockdown in human CRC cells, which showed the increased expression of granzyme B (GZMB) in TRIP13 knockdown cells (fig. 7A of Agarwal et al., [11]). This relation was validated in CRC cells by western blot analysis indicating that TRIP13 inhibition could be leading to increased GZMB, a marker of T‐cell infiltration (Fig. 6A). However, immunocompetent model is an ideal setting to investigate whether DCZ0415 could enhance an *in vivo* antitumour immune response. Therefore, we used a syngeneic mouse model to study infiltration of immune cells after DCZ0415 treatment. We injected murine CRC MC38 cells, which exhibit TRIP13 expression (Fig. 6H), into C57/BL6 mice and treated them with DCZ0415 (25 mg·kg^−1^) or vehicle (Fig. 6B). DCZ0415 treatment suppressed tumour growth, as shown by reduced tumour weight (Fig. 6C) and size (Fig. 6D,E) in the DCZ0415‐treated group compared to those in the vehicle control group. Further, we investigated the levels of T‐cell infiltration in tumour tissues using IHC analysis that showed the immune infiltration of CD3, CD4 and CD8 in DCZ0415‐treated tumours indicating that the infiltration of T cells could be leading to tumour regression in mice (Fig. 6F). Moreover, RNA levels of PD1 and CTLA4 were low in DCZ0415‐treated tumours as compared to tumours of mice treated with vehicle (Fig. 6G). However, only PD1 expression levels were significantly low (*P* = 0.03). Furthermore, low RNA expression levels of PD1 were reflected in decreased protein levels (Fig. 6H) in DCZ0415‐treated tumours suggesting that DCZ0415 treatment could be activating an antitumour immune response by targeting these two immune checkpoints and leading to less tumour growth in this immunocompetent model. Furthermore, western blot analysis showed increased levels of GZMB in DCZ0415‐treated tumours (Fig. 6H), validating our initial observation that TRIP13 inhibition increases levels of GZMB, indicating increased T‐cell infiltration. In addition, the levels of cytotoxic mediators, perforin and IFN‐γ, were found to be increased (Fig. 6H), indicating the activation of antitumour immune response in this syngeneic model after treatment with DCZ0415. Therefore, these data suggest that DCZ0415 treatment could enhance the anticancer immunity.

## 4 Discussion

TRIP13 is frequently upregulated in many cancers [4, 26, 43, 44, 45], including CRCs [12, 13, 14, 15]. Our previous study provided evidence that TRIP13 contributes to the progression of CRCs and suggested that inhibition of TRIP13 activation could be useful for the treatment of CRC [11]. As TRIP13 has a role in promoting CRC progression, we tested the hypothesis that pharmacologic inhibition of TRIP13 by DCZ0415 inhibits primary and metastatic tumour growth. The effects of DCZ0415 were evaluated *in vitro* and *in vivo* to determine changes in the malignant phenotype of CRC.

Our results indicated that DCZ0415 inhibited malignant phenotypes of CRC cells and showed that DCZ0415 treatment caused cell cycle G2/M phase arrest and decreased cell numbers in the G0/G1 phase. Furthermore, induction of apoptosis was verified using Annexin V‐FITC/PI apoptosis detection assay, to discriminate living cells from early and late apoptosis. Our results also indicate a decrease in full‐length PARP protein, an indicator of apoptotic cell death, when CRC cells treated with DCZ0415. The antitumour activity of DCZ0415 was further supported by animal experiments. Specifically, immunocompromised NSG mice with CRC xenograft tumours treated with DCZ0415 developed smaller tumours, showing that inhibition with DCZ0415 compromised the growth of CRCs. Likewise, preclinical studies of the effects of DCZ0415 on multiple myeloma xenografts or immunocompromised models demonstrate inhibition of tumour growth [26].

In epithelial tumours, the EMT is involved in invasion and metastasis [46]. Our prior study showed that TRIP13 knockdown reduces mesenchymal phenotypes and increases epithelial features of the EMT pathway [11]. In addition, a previous study demonstrates that TRIP13 and YWHAZ, a member of the 14‐3‐3 family of proteins, are positively related in CRC and modulate the EMT in an YWHAZ‐dependent way [12]. Similarly, our current study showed that DCZ0415 treatment modulated the EMT pathway, indicated by an increased expression of E‐cadherin while decrease in the expression of N‐cadherin and Snai1. In xenografts, N‐cadherin levels were lower in tumours after DCZ0415 treatment. This could be due to physical interaction of FGFR4 with N‐cadherin [38, 47], and this interaction could have been breached by DCZ0415 treatment. A recent study also showed the attenuation of EMT in perihilar cholangiocarcinoma cells treated with DCZ0415 [16]. These data indicate that DCZ0415 inhibits TRIP13‐mediated CRC progression through blocking the EMT.

Our previous study shows an interaction between FGFR4 and TRIP13 and suggests that this interaction could be relevant for CRC progression [11]. Moreover, FGFR4 acts through phosphorylation of STAT3, a target of FGFR4 [48]. Thus, we hypothesized that exposure to DCZ0415 affects FGFR4 and its downstream oncogenic STAT3 activation. We showed that, for multiple CRC cell lines, DCZ0415 treatment decreased the levels of FGFR4. In addition to TRIP13 involvement in modulating FGFR4 expression in CRC, we showed, by knockdown studies, that TRIP13 activates STAT3, which is a signalling mechanism for the growth and progression of CRC. Decreased activation of STAT3 through inhibiting TRIP13 by DCZ0415, observed with *in vitro* cultures and *in vivo* xenograft models, suggested that the antitumour activity of DCZ0415 was due to inactivation of the FGFR4–STAT3 axis. These results align with findings in the context of FGFR4 knockdown, which show lower activity of STAT3 after FGFR4 silencing in CRC cells [48].

Further, the interaction between NF‐κB and STAT3 contributes to tumourigenesis in various cancers via induction of molecules that are involved in hypoxia and angiogenesis, and by induction of chemokines and cytokines [39, 40]. Our findings also showed that, in DCZ0415‐treated cells, phosphorylation of NF‐κBp65 decreased along with the inactivation of STAT3 and decreased levels of FGFR4. This suggests that DCZ0415 represses NF‐κB activity through the inactivation of the STAT3–FGFR4 axis. Therefore, it reasonable to propose that an increase in TRIP13 levels leads to CRC progression through hyper‐activation of FGFR4/STAT3/NF‐κB, which can be inhibited by DCZ0415. In the current study, the evaluation of human CRC tissues revealed a positive correlation between the expression of TRIP13 and FGFR4, suggesting a link between these two oncogenes. This study also demonstrated that TRIP13 regulates the expression of FGFR4 and activates its STAT3 and NF‐kB pathways, which are involved in the aggressiveness of CRCs.

The stimulation of the FGFR4 pathway endows cancers with the capacity to resist chemotherapy [49]. FGFR4 overexpression increases Bcl‐x expression through the MAPK cascade, implying that FGFR4 inhibitors combined with chemotherapeutic drugs could be useful for treating FGFR4‐overexpressing cancers [50]. Drug‐resistant cells activate FGFR4 signalling to phosphorylate FGF receptor substrate 2 (FRS2) and then activate downstream MAPK/ERK signalling. Inhibitors that block FGFR4–FRS2–ERK signalling restrain the glycolytic phenotypes and chemoresistance of resistant cells [51]. Futibatinib, a potent, selective and irreversible small‐molecule inhibitor of FGFR1/2/3/4, inhibits the growth of FGFR abnormal cells and tumour xenografts [52], shows positive responses in trials of patients with advanced solid tumours [53] and is approved for patients with advanced intrahepatic cholangiocarcinomas that exhibit abnormal FGFR and who have failed at least one line of therapy (www.fda.gov/drugs). Since TRIP13 activates FGFR4, and DCZ045 specifically inhibits TRIP13, future studies should focus on combining DCZ0415 with futibatinib, an FDA‐approved inhibitor of FGFR that has antitumour activity [52]. Thus, future trials should be conducted by combining FGFR4 inhibitor with DCZ0415 to improve the response to DCZ0415.

In CRCs, there is an abnormal activation of the Wnt/β‐catenin pathway, and its activation increases β‐catenin protein in the nucleus, which forms complexes with TCF/LEF to regulate target gene expression [54]. Since our previous report indicates that TRIP13 is involved in the Wnt/ β‐catenin signalling pathway [11], we checked for the expression of molecules involved in this pathway in DCZ0415‐treated cell and xenograft lysates, which indicated the inactivation of this pathway with DCZ0415 treatment. Moreover, our analyses of clinical specimens confirmed a positive correlation between elevated TRIP13 and an activated Wnt/β‐catenin pathway. Thus, our findings suggest that TRIP13‐mediated Wnt/β‐catenin activation can be inhibited by DCZ0415.

Increased tumour‐infiltrating CD8+ T cells are considered the major effector immune cells in antitumour immunity. Granzyme B and perforin are important cytotoxic constituents of natural killer cells and cytotoxic T lymphocyte cells to mediate apoptosis in tumour cells [55]. A prior study reported that DCZ0415 significantly increases the infiltration of CD3, CD4 and CD8 in a myeloma model [26]. Our findings of RNA sequence data have revealed the activation of granzyme B in TRIP13 knockdown cells. Further, we validated this observation in an immunocompetent mouse CRC model, which demonstrated that DCZ0415 substantially increases the secretion of cytotoxic mediators, granzyme B, perforin and IFN‐γ that may contribute to the observed cytotoxic action against murine MC38 cells. Additionally, we noted lower levels of PD1 and CTLA4 suggesting a possible blockade of these immune checkpoints in DCZ0415‐treated tumours that improved cytotoxicity and tumour regression. It was previously shown that STAT3 is central in regulating the antitumour immune response by inhibiting the expression of crucial immune activation regulators (IL6 and IL10), promoting the production of immunosuppressive factors (Treg cells), increasing the expression of immune checkpoint molecules (PD1 and CTLA4) and reducing the antitumourigenic effector functions of CD8^+^ T cells [56]. Our study showed that DCZ0415 inactivates STAT3, which could be a reversing primary function of STAT3, leading to anticancer immunity and reduced MC38 tumours. The findings support that DCZ0415 could be used as an immune stimulatory molecule in the treatment of cancer.

## 5 Conclusion

In summary, the data presented here illustrate that, for a subset of colorectal tumours exhibiting TRIP13 overexpression, TRIP13 as an attractive therapeutic target. Our results showed a link between high levels of TRIP13 and overexpression of FGFR4 in CRCs. Furthermore, our results demonstrate that TRIP13 inhibition by DCZ0415 reduces tumour growth and metastasis, irrespective of *p53, KRAS, BRAF*, EGFR and MSI status of CRC cells, by blocking the EMT and WNT/β‐catenin pathways. DCZ0415 treatment decreased cell proliferation, colony formation, invasion, migration, tumour growth and metastasis. Treatment with DCZ0415 inhibited the EMT process, and decreased the activation of the NF‐κB and Wnt/β‐catenin signalling pathways. In CRC cells, DCZ0415 also caused a G2/M phase arrest and induced apoptosis through inactivation of the FGFR4/STAT3/NF‐κB axis. Additionally, DCZ04145 treatment activates antitumour immune response by upregulating cytotoxic mediators. Our findings suggest that inhibition of TRIP13 by DCZ415 is a potential therapeutic strategy for a subset of CRCs, those that exhibit overexpression of TRIP13 and FGFR4. The data provide a basis for conducting a biomarker‐driven clinical trial to target TRIP13 in CRC.

## Conflict of interest

The authors declare no conflict of interest.

## Author contributions

SA: Conceptualization, investigation, methodology, data curation, formal analysis and writing–original draft. FA: Investigation, data curations and manuscript editing. PB: Investigation and data curations. H‐GK: Investigation. AE: Investigation. MB: Bioinformatic and statistical analysis. DSC: Bioinformatic analysis. SAD: Analysed histopathology and IHC evaluations. MK: Resources. SPS: Resources. SV: Conceptualization, resources and manuscript editing. UM: Conceptualization, methodology, resources, funding acquisition, project administration, writing–reviewing and editing.

### Peer review

The peer review history for this article is available at https://publons.com/publon/10.1002/1878‐0261.13201.

## Supporting information


**Fig. S1**. DCZ0415 decreases invasion and migration of CRC cells.

**Fig. S2**. mRNA expression of EMT markers in CRC cells.

**Table S1**. shRNA sequences used in this study—related to Materials and methods.
**Table S2**. List of antibodies used in this study—related to Materials and methods.
**Table S3**. qPCR primer sequences used in this study— related to Materials and methods.

## Data Availability

The data presented in this study are available from the corresponding author on reasonable request.
