# Supplementary figures and images for: DCZ0415, a small‐molecule inhibitor targeting TRIP13, inhibits EMT and metastasis via inactivation of the FGFR4/STAT3 axis and the Wnt/β‐catenin pathway in colorectal cancer
Source: Mol Oncol. 2022 Mar 7;16(8):1728–45. doi: 10.1002/1878-0261.13201 (PMC9019876; doi:10.1002/1878-0261.13201)

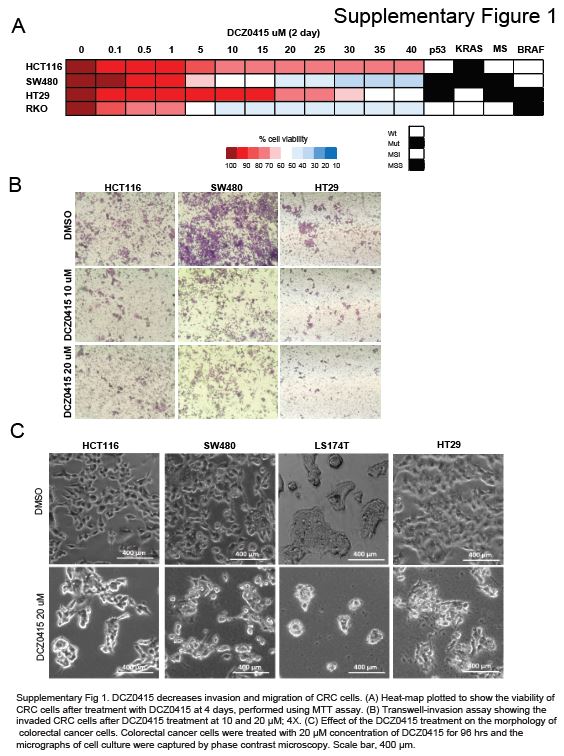

Supplement: Supplementary file 1 — Fig. S1. DCZ0415 decreases invasion and migration of CRC cells. [file MOL2-16-1728-s001.jpg]

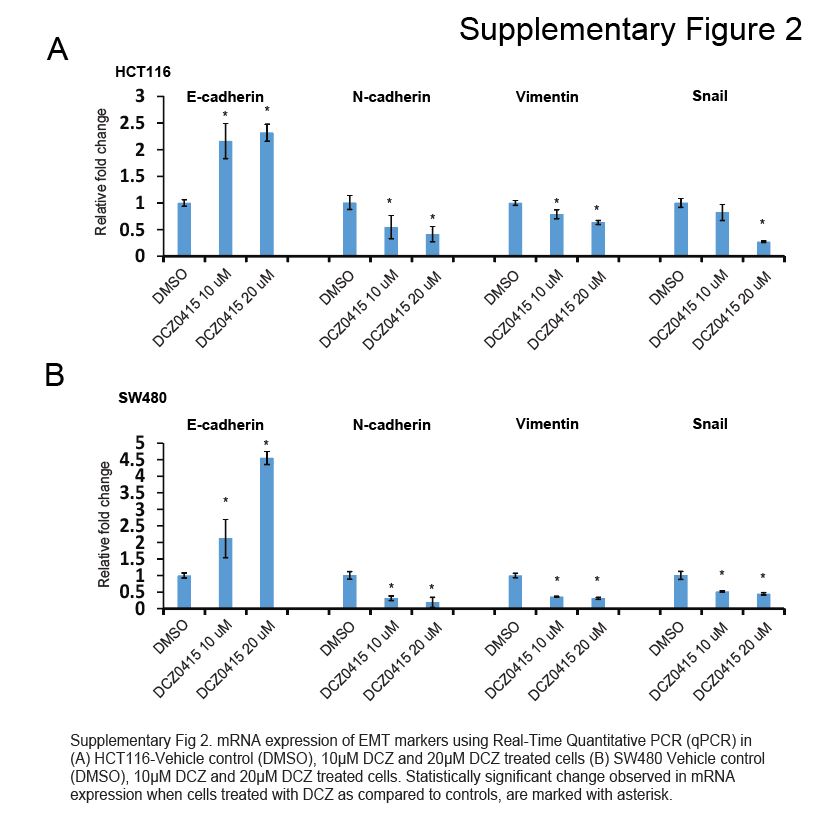

Supplement: Supplementary file 2 — Fig. S2. mRNA expression of EMT markers in CRC cells. [file MOL2-16-1728-s002.jpg]
