# Supplementary material for: DCZ0415, a small‐molecule inhibitor targeting TRIP13, inhibits EMT and metastasis via inactivation of the FGFR4/STAT3 axis and the Wnt/β‐catenin pathway in colorectal cancer
Source: Mol Oncol. 2022 Mar 7;16(8):1728–45. doi: 10.1002/1878-0261.13201 (PMC9019876; doi:10.1002/1878-0261.13201)
Supplement: Supplementary file 3 — Table S1. shRNA sequences used in this study—related to Materials and methods. Table S2. List of antibodies used in this study—related to Materials and methods. Table S3. qPCR primer sequences used in this study— related to Materials and methods. [file MOL2-16-1728-s003.docx]

**DCZ0415, a small-molecule inhibitor targeting TRIP13, inhibits the EMT and metastasis via inactivation of the FGFR4/STAT3 axis and the Wnt/β-catenin pathway in colorectal cancer**

Sumit Agarwal^1^, Farrukh Afaq^1^, Prachi Bajpai^1^, Hyung-Gyoon Kim^1^, Amr Elkholy^1^, Michael Behring^1^, Darshan Shimoga Chandrashekar^1^, Sameer Al Diffalha^1,2^, Moh’d Khushman^2,3^, Shajan P. Sugandha^4^, Sooryanarayana Varambally^1,2^, Upender Manne^1,2*^

^1^Department of Pathology, University of Alabama at Birmingham

^2^O’Neal Comprehensive Cancer Center, University of Alabama at Birmingham, Birmingham, AL

^3^Department of Medicine, Division of Medical Oncology, University of Alabama at Birmingham

^4^Department of Medicine, Division of Gastroenterology, University of Alabama at Birmingham, AL 35233, USA

^*^**Correspondence**: Upender Manne, MS, PhD, Professor and Director of Translational Anatomic Pathology, Wallace Tumor Institute, Room # 420A, University of Alabama at Birmingham, Birmingham, AL 35233, USA

Phone: (205)-934-4276, Email: [upendermanne@uabmc.edu](mailto:upendermanne@uabmc.edu)

**Running Title:** DCZ0415 reduces metastasis in colorectal cancer

**Supplementary Tables, Related to Methods**

Keywords: Colorectal cancer; TRIP13; DCZ0415; FGFR4; metastasis; granzyme B

**SUPPLEMENTARY TABLES**

**Table S1.** shRNA sequences used in this study, Related to Materials and Methods.

| **Gene name** | **Catalog No** | **Supplier** | **Sequence** |
| --- | --- | --- | --- |
| **TRIP13** | shRNA 1  shRNA 2 | System Biosciences, Mountain View, CA | GUACCGAUAUGGCCAAUUA  GCAAAUCACUGGGUUCUAC |

**Table S2.** List of antibodies used in this study, Related to Materials and Methods.

| **Antibody** | **Application** | **Dilution** | **Supplier** | **Cat. No.** |
| --- | --- | --- | --- | --- |
| TRIP13 | IB | IB, 1:1000 | PTG Labs, Chicago, IL | 19602-1-AP |
| Cyclin D1 | IB | IB, 1: 1,000 | Cell Signaling Technology, Danvers, MA | 2978P |
| LEF1 | IB | IB, 1:1000 | Cell Signaling Technology, Danvers, MA | 2230P |
| β-Catenin | IB | IB, 1:1000 | Cell Signaling Technology, Danvers, MA | 9562 |
| EGFR-Y-1068 | IB | IB, 1:1000 | Cell Signaling Technology, Danvers, MA | 3777S |
| Total-EGFR | IB | IB, 1:1000 | Cell Signaling Technology, Danvers, MA | ab131498 |
| β –Actin-HRP | IB | IB, 1:10000 | PTG Labs, Chicago, IL | HRP-60008 |
| FGFR4 | IB | IB, 1:1000 | Cell Signaling Technology, Danvers, MA | 8562 |
| p-STAT3 Y705 | IB | IB, 1:1000 | Cell Signaling Technology, Danvers, MA | 9145S |
| STAT3 |  |  |  |  |
| N-Cadherin | IB | IB, 1:1000 | Proteintech, Chicago, IL | 22018-1-AP |
| Snail | IB | IB, 1:1000 | Cell Signaling Technology, Danvers, MA | 3879P |
| Phospho-NF-κB p65 (Ser536) (93H1) | IB | IB, 1:1000 | Cell Signaling Technology, Danvers, MA | 3033S |
| Phospho-IKKα/β (Ser176/180) (16A6) | IB | IB, 1:1000 | Cell Signaling Technology, Danvers, MA | 2697 |
| Granzyme B | IB | IB, 1:1000 | PTG Labs, Chicago, IL | 13588-1-AP |
| Perforin | IB | IB, 1:1000 | PTG Labs, Chicago, IL | 14580-1-AP |
| IFN gamma | IB | IB, 1:1000 | PTG Labs, Chicago, IL | 15365-1-AP |
| PD-1 | IB | IB, 1:1000 | PTG Labs, Chicago, IL | 18106-1-AP |
| E-cadherin | IB | IB, 1:1000 | Cell Signaling Technology, Danvers, MA | 3195T |
| PARP | IB | IB, 1:1000 | Cell Signaling Technology, Danvers, MA | 9542T |
| PCNA | IB | IB, 1:1000 | Proteintech, Chicago, IL | 1020052-2-AP |
| Anti-Rabbit IgG HRP | IB | IB, 1:5000 | PTG Labs, Chicago, IL | SA00001-2 |
| Anti-Mouse IgG HRP | IB | IB, 1:5000 | PTG Labs, Chicago, IL | SA00001-1 |

IB: Immunoblotting

**Table S3.** qPCR Primer sequences used in this study, Related to Materials and Methods.

| **Gene name** | **Forward primer** | **Reverse primer** |
| --- | --- | --- |
| ***PD-1*** | TCATGAGTGCCCTAGTGGGT | TCCTCTGGCCTCTGACATACT |
| ***CTLA-4*** | CTGCAGCTGCCTTCTAGGAC | TTGGGTCACCTGTATGGCTTC |
| ***ACTB*** | GCACAGAGCCTCGCCTT | GTTGTCGACGACGAGCG |
| ***E-cadherin*** | AAGAAGCTGGCTGACATGTACGGA | CCACCAGCAACGTGATTTCTGCAT |
| ***N-cadherin*** | TGTGGGAATCCGACGAATGGATGA | TGGAGCCACTGCCTTCATAGTCAA |
| ***Snai1*** | TGCCCTCAAGATGCACATCCGA | GGGACAGGAGAAGGGCTTCTC |
| ***Vimentin*** | AGAACCTGCAGGAGGCAGAAGAAT | TTCCATTTCACGCATCTGGCG TT |
| ***β-actin*** | GCACAGAGCCTCGCCTT | GTTGTCGACGACGAGCG |
